# Association of Human Whole Blood NAD^+^ Contents With Aging

**DOI:** 10.3389/fendo.2022.829658

**Published:** 2022-03-21

**Authors:** Fan Yang, Xuan Deng, Ye Yu, Lei Luo, Xianda Chen, Jinping Zheng, Yugang Qiu, Feng Xiao, Xiaomei Xie, Yuzheng Zhao, Jun Guo, Feifei Hu, Xuguang Zhang, Zhenyu Ju, Yong Zhou

**Affiliations:** ^1^ The First Affiliated Hospital of Jinan University, Institute of Aging and Regenerative Medicine, Jinan University, Guangzhou, China; ^2^ Clinical Research Institute, Shanghai General Hospital, Shanghai Jiao Tong University School of Medicine, Shanghai, China; ^3^ Administrative Office, Total Quality Management Office, Total Quality Management Institute, Shanghai General Hospital, Shanghai Jiao Tong University School of Medicine, Shanghai, China; ^4^ Department of Public Health and Preventive Medicine, Changzhi Medical College, Changzhi, Shanxi, China; ^5^ School of Rehabilitation Medicine, Weifang Medical University, Weifang, China; ^6^ Tangshan Gem Flower Hospital, Tangshan, China; ^7^ Optogenetics & Synthetic Biology Interdisciplinary Research Center, State Key Laboratory of Bioreactor Engineering, Shanghai Frontiers Science Center of Optogenetic Techniques for Cell Metabolism, School of Pharmacy, East China University of Science and Technology, Shanghai, China; ^8^ BYHEALTH Institute of Nutrition & Health, Guangzhou, China

**Keywords:** Nicotinamide adenine dinucleotide, whole blood NAD^+^, aging, gender-difference, NAD^+^

## Abstract

**Background:**

NAD^+^, nicotinamide adenine dinucleotide, is mostly described to associate with the aging process. We aimed to investigate the association between human whole blood NAD^+^ contents and aging in a relative large-scale community-based population and further to address the gender impact on this association.

**Methods:**

We recruited 1,518 participants aged over 18 years old and free of cardiovascular and any type of cancer from the Jidong community from 2019 to 2020. Whole blood NAD^+^ level was measured by cycling assay and LC-mass spectroscopy assay. The chronological age and clinical data were collected using standard questionnaires. The participants were divided into five groups according to their chronological age. General liner regression model was performed to analyze the association between NAD^+^ contents and aging. In addition, we also conducted subgroup analysis by gender.

**Results:**

The mean age of included 1,518 participants was 43.0 years, and 52.6% of them were men. The average levels of whole blood NAD^+^ of total participants was 33.0 ± 5.5 μmol/L. The whole blood NAD^+^ contents in men were significantly higher than that in women (34.5 vs. 31.3 μmol/L). There was significant difference in the meat diet among NAD^+^ quartile groups (p = 0.01). We observed a decline trend of NAD^+^ contents with aging before 50 years in total participants with significant level in 40–49 years old group (β coefficients with 95% confidence interval (95% CI): −1.12 (−2.18, −0.06)), while this trend disappeared after the 50 years. In addition, this association was significantly altered by gender (p for interaction = 0.003). In men, as compared with ≤29 years group, adjusted β coefficient decreased with aging but was only significant in the ≥60 year group (β,−2.16; 95% CI, −4.16 to −0.15). In females, the level of whole blood NAD^+^ did not significantly differ among five age groups and without the trend as males.

**Conclusions:**

Association of whole blood NAD^+^ contents with aging significantly differed in males and females. The loss of blood NAD^+^ with aging only was observed in males, especially in the male middle-aged population. It is crucial to consider the gender difference in further NAD^+^ related studies in the future.

## Introduction

Aging is characterized by a progressive decline of energy metabolism and physiological function, which serves as a major risk factor for several diseases, namely, cancer, cardiovascular disease, and neurodegenerative disease (1–4). The molecular mechanism behind aging is still one of the most notable subjects in science. Nicotinamide adenine dinucleotide (NAD^+^), a multifunctional metabolite, is mostly described as a major player in aging-related diseases (5–8). NAD^+^ levels are maintained by three independent pathways, namely, the Preiss–Handler pathway, the *de novo* synthesis pathway, and the NAD^+^ salvage pathway (9, 10). NAD^+^ is an important co-substrate for the Sirtuins, PARPs, and the cyclic ADP-ribose (cADPR) synthases (11). It involves in the process of oxidative reductive reactions of the cellular respiration processes and regulating the activities of the NAD^+^-consuming enzymes to mediate the cellular signaling transduction (5, 12). Declined blood NAD^+^ content in human was related to aging in a half of studies but not in the other half of researches (13–15). A recent study had reported the age-related decline of NAD^+^ might be caused by the hyperactivity of NAD^+^ consuming enzymes (16). Gomes et al. demonstrated that a lack of NAD^+^ production significantly contributes to aging, which can be ameliorated by boosting NAD^+^ production with nicotinamide mononucleotide (17). However, it is still unclear whether or not whole blood NAD^+^ contents are associated with aging. Moreover, the physiological relevance of whole blood NAD^+^ to predict aging-associated conditions clearly needs further investigation. Thus, monitoring basal NAD^+^ levels in human could help us understand the mechanism of aging and aging-related diseases leading the way to find potential treatments. Furthermore, the sample size in human studies was relatively small (13, 14, 18). For addressing the specific concern, it needs relatively large-scale epidemiological studies to minimize selection bias.

In addition, it has been reported that the sex differences exist in NAD^+^ baseline levels and stroke-induced loss of NAD^+^ in mice (19). A study also proposed that there is the sex-difference in prolonging the life span of mice after enhancing expression of NAMPT which is a key rate-limiting enzyme in the NAD^+^ synthesis pathway (20). Hence, we speculated that the association of aging and NAD^+^ might also be modified by gender. Nevertheless, up to now, there is very limited evidence on the gender differences in human NAD^+^ related studies. In this study, we aimed to address the change of NAD^+^ levels in human whole blood with aging in a relatively large-scale community-based population and further characterize the gender impact on the association of whole blood NAD^+^ contents with aging.

## Methods

### Study Design and Population

**Figure 1:**
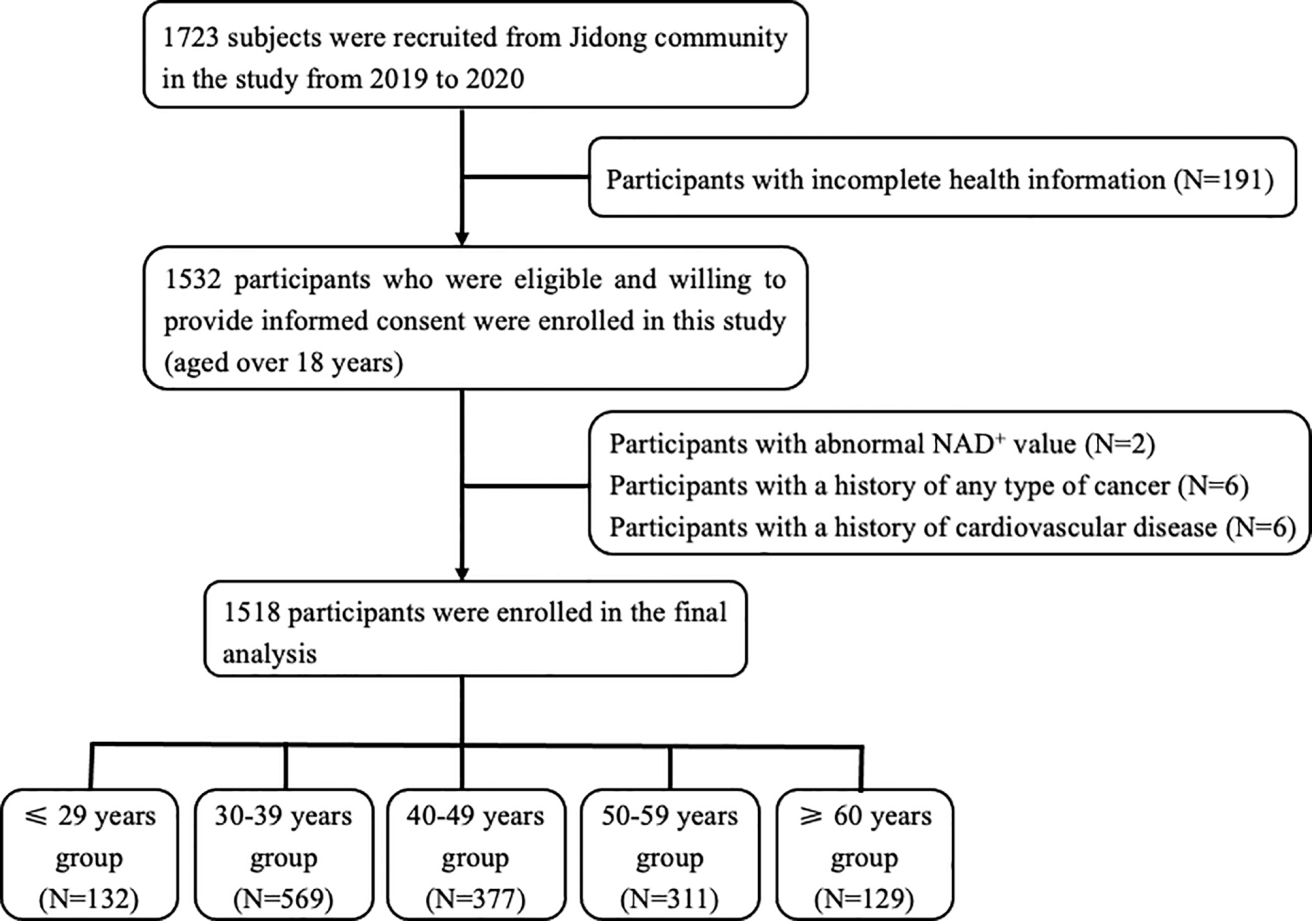
Flow chart of the enrolled participants.

In this cross-sectional study, the participants were recruited from the Jidong community located in Caofeidian district, Tangshan city, northern China from 2019 to 2020. Of the 1,723 participants randomly selected, 1,532 had complete health information and agreed to provide informed consent for measuring the level of NAD^+^ in whole blood. Among these participants, 14 subjects were excluded by the following exclusion criteria: [1] presence of cardiovascular disease, namely, atrial fibrillation, heart failure, myocardial infraction, and coronary heart disease; [2] presence of any type of cancer history; and [3] participants with abnormal NAD^+^ value. Given that vitamin B affects the NAD^+^ metabolism, we collected data on the vitamin intake history in the questionnaire. Among the participants included, there was no one who had history of vitamin intake. Ultimately, a total of 1,518 participants were included in the analysis (**Figure 1**). The study was performed according to the principle of the Helsinki Declaration and the study protocol was approved by the Ethical Committees of the Staff Hospital of Jidong Oilfield of China National Petroleum Corporation.

### Baseline Data Collection

Baseline data was collected by well-trained research coordinators through standardized questionnaires. The baseline data included participants demographics, lifestyles, medical conditions, and other relevant factors. The medical conditions included hypertension, diabetes mellitus, and hyperlipidemia, which were defined according to [1] documented or self-reported history or [2] receiving medication for corresponding diseases or [3] clinical or laboratory examination [blood pressure ≥140/90 mmHg on repeated measurements for a diagnosis of hypertension; fasting blood glucose level ≥7.0 mmol/L for diabetes mellitus; serum total cholesterol ≥5.7 mmol/L, triglyceride≥1.7 mmol/L, low-density lipoprotein cholesterol level≥4.1 mmol/L for hyperlipidemia (21)]. Lifestyles included the history of smoking and alcohol consumption and body mass index (BMI). BMI was calculated by dividing measured weight (kg) by the square of measured height (m^2^) and was categorized as “<18.5”, “18.5–23.9”, “24.0–27.9”, and “>28.0”. The daily diet included the intake frequency of meat and vegetable were defined as “Never”, “Occasionally”, and “Very often”. In addition, due to the number of red blood cells (RBC) could affect the results of NAD^+^ measurement, we also recorded the number of RBC which was tested by autoanalyzer (Hita’chi 747; Hitachi, Tokyo, Japan).

### Testing for Whole Blood NAD^+^

Blood samples were collected from the large antecubital veins after overnight fasting. All blood samples were collected into vacuum tubes containing EDTA (ethylene diamine tetraacetic acid), and NAD^+^ levels were determined by the cycling assay and LC–MS/MS analysis in the laboratories. Every sample was separated into two parts for two methods.

For cycling assay, the blood samples were extracted with 0.5M HClO_4_ immediately after collection, then centrifuged at 10,000×*g* for 15 min. Organic solvent (1,1,2-trichloro-1,2,2-trifluoroethane: Trioctylamine = 3:1) was added to remove HClO_4_ at a ratio of 2:1. We carefully removed the top aqueous layer containing the NAD^+^ and added 1 M Tris to adjust the pH to 8.0. Approximately 100 ul supernatants were mixed with 100 ul reaction medium containing CCK-8, 13 units/ml alcohol dehydrogenase, 100 mM nicotinamide, 5.7% ethanol in 61 mM Gly-Gly buffer (pH 7.4). Samples were mixed in a 96-well plate samples at room temperature. The A450 nm was determined immediately after 30 min, and results were calibrated with NAD^+^ standards. The total NAD^+^ was quantified using a plate reader. Because of experiment variation in sample processing, samples were processed in parallel. The normalized values from each experiment were used to obtain the presented average values.

For LC–MS/MS analysis, the blood samples were extracted with 0.5 M HClO_4_, and then centrifuged at 10,000×*g* for 15 min. Organic solvent (1,1,2-trichloro-1,2,2-trifluoroethane: Trioctylamine = 3:1) was added to remove HClO_4_ at a ratio of 2:1. Then the top aqueous layer containing the NAD^+^ was carefully removed for LC–MS/MS analysis. Stock solution preparation: NAD^+^ (1 mM in water) and ^13^C_5_-NAD^+^ (IS, 1 mM in water) were stored at −80 °C. Working standards were prepared by diluting above stocks in water containing 500 nM internal standard. Blood samples: Samples were diluted in HClO_4_ containing 500 nM of internal standard. Samples were transferred to sample vials for immediate analysis. NAD^+^ analysis in blood samples was carried out with an Agilent 1260 couples to a QTRAP@ 4500 mass spectrometer (AB SCIEX) equipped with an electrospray ionization source. The aqueous mobile phase was water with ammonium acetate (A) and the organic mobile phase was methanol (B) with a constant flow rate of 0.4 ml/min and a total run time of 11 min. The elution was initiated at 96% A and held for 1.5 min, then B was increased to 100% for 2.5 min, and returned to initial conditions in 4.5min. Auto-sampler temperature was 22–25 °C and sample injection volume was 5 μl and NAD^+^ was eluted at 3.19 min. Detection of NAD^+^ was accomplished using the mass spectrometer in negative ESI mode using capillary voltage 4.5 kV, source temp 400 °C, desolvation gasI flow 55 psi and gasII flow 50 psi. NAD^+^ quantitation was carried using the multiple reaction monitoring (MRN) transition m/z 662.1>540.0 for NAD^+^ and m/z 667.1>545.1 for the internal standard (^13^C_5_-NAD^+^). Data was acquired and analyzed by SCIEX v1.6.3 software. The reliability of the NAD^+^ levels obtained with the cycling assay was verified by comparing the data with those determined by conventional LC–MS/MS (**Figure S1**).

### Participant Grouping According to Age

Initially, the participants were grouped according to their chronological age by ten years: ≤29, 30–39, 40–49, 50–59, 60–69, and ≥70 years. Considering the small sample of the ≥70 years group, the groups were then divided into following groups: ≤29, 30–39, 40–49, 50–59, and ≥60 years.

### Statistical Analysis

The Kolmogorov–Smirnov test was performed to test the normality of the continuous variables. The continuous variables were described as the mean ± standard deviation (SD) and the categorical variables were expressed as counts (percentages). One-way analysis of variance (ANOVA) was performed to test the difference among the age groups for continuous variables, the chi-square test and Fisher test was performed for categorical variables. The general liner regression model was used to analyze the associations between the level of NAD^+^ in blood and aging process. In addition, the stratification analysis was made by gender. The following factors were adjusted in the regression models: BMI, smoking and drinking history, daily diet, the history of hypertension, hyperlipidemia, and diabetes, and the number of RBC. Associations were measured as β coefficients with 95% confidence intervals (CIs). Two-sided p <0.05 is considered to be statistically significant. Analysis was performed by using SAS software, version 9.4 (SAS Institute Inc., Cary, North Carolina, USA).

## Results

### Participant Characteristics

Characteristics of the study population are summarized in **Table 1**. Of the included 1,518 participants, the average age was 43.0 years, and 52.6% were men. The average whole blood level of NAD^+^ was 33.0 ± 5.5 μmol/L in total participants. There were significant differences among five age groups in gender, BMI, smoking and drinking history, and the history of hypertension, diabetes, and hyperlipidemia (p <0.01 for gender, and p <0.001 for other variables). The prevalence of hypertension, hyperlipidemia, and diabetes increased with aging. However, the level of NAD^+^ did not significantly differ among five groups in total population. In addition, we found there was significant difference in meat diet among NAD^+^ quartile groups (p = 0.01), but not in vegetable diet (p = 0.81) (**Supplementary Table 1**). However, a significant correlation was not observed between NAD^+^ quartiles and meat diet by conducting Spearman analysis (r = 0.20, p = 0.429).

**Table 1:** Baseline characteristics of the study population.

Characteristics	Age groups (years)						p-value
	Total	≤29	30–39	40–49	50–59	≥60	
N, %	1,518	132 (8.7)	569 (37.5)	377 (24.8)	311 (20.5)	129 (8.5)	
Males (N, %)	798 (52.6)	57 (43.2)	307 (54.0)	185 (49.1)	183 (58.8)	66 (51.2)	0.02
BMI (kg/m^2^)							<0.001
<18.5	160 (10.5)	24 (18.2)	75 (13.2)	36 (9.6)	18 (5.8)	7 (5.4)	
18.5–23.9	587 (38.7)	57 (43.2)	233 (41.0)	140 (37.1)	111 (35.7)	46 (35.7)	
24.0–27.9	551 (36.3)	26 (19.7)	184 (32.3)	152 (40.3)	133 (42.8)	56 (43.4)	
>28.0	220 (14.5)	25 (18.9)	77 (13.5)	49 (13.0)	49 (15.8)	20 (15.5)	
Education level (N, %)							<0.001
Middle school or below	358 (23.6)	12 (9.1)	44 (7.7)	93 (24.7)	126 (40.5)	83 (64.3)	
College or above	1,160 (76.4)	120 (90.9)	525 (92.3)	284 (75.3)	185 (59.5)	46 (35.7)	
Smoking history (N, %)							0.004
Never	1,147 (75.6)	110 (83.3)	437 (76.8)	279 (74.0)	215 (69.1)	106 (82.2)	
Current & Former	371 (24.4)	22 (16.7)	132 (23.2)	98 (26.0)	96 (30.9)	23 (17.8)	
Drinker (N, %)	265 (17.5)	13 (9.9)	89 (15.6)	83 (22.0)	68 (21.9)	12 (9.3)	<0.001
Hypertension (N, %)	426 (28.1)	14 (10.6)	87 (15.3)	108 (28.7)	143 (46.0)	74 (57.4)	<0.001
Diabetes (N, %)	123 (8.1)	4 (3.0)	22 (3.9)	34 (9.0)	40 (12.9)	23 (17.8)	<0.001
Hyperlipidemia (N, %)	797 (52.5)	50 (37.9)	252 (44.3)	216 (57.3)	200 (64.3)	79 (61.2)	<0.001
RBC (10^12^/L)	4.76 ± 0.50	4.80 ± 0.54	4.78 ± 0.52	4.72 ± 0.50	4.76 ± 0.46	4.70 ± 0.40	0.19
NAD^+^ (μmol/L)	33.00 ± 5.54	33.33 ± 5.07	33.14 ± 5.77	32.51 ± 5.50	33.31 ± 5.45	32.71 ± 5.28	0.28
Meat diet (N, %)							0.66
Never	32 (2.1)	1 (0.8)	9 (1.6)	10 (2.7)	9 (2.9)	3 (2.3)	
Occasionally	1,222 (80.5)	106 (80.3)	464 (81.6)	299 (79.3)	244 (78.5)	109 (84.5)	
Very often	264 (17.4)	25 (18.9)	96 (16.9)	68 (18.0)	58 (18.7)	17 (13.2)	
Vegetable diet (N, %)							0.71
Never & Occasionally	100 (6.6)	8 (6.1)	34 (6.0)	30 (8.0)	18 (5.8)	10 (7.8)	
Very often	1,418 (93.4)	124 (93.9)	535 (94.0)	347 (92.0)	293 (94.2)	119 (92.3)	

Values for categorical variables are given as number or number (percentage); Values for continuous variables are given as mean ± standard deviation.

### NAD^+^ Whole Blood Level Depending on the Gender

**Figure 2:**
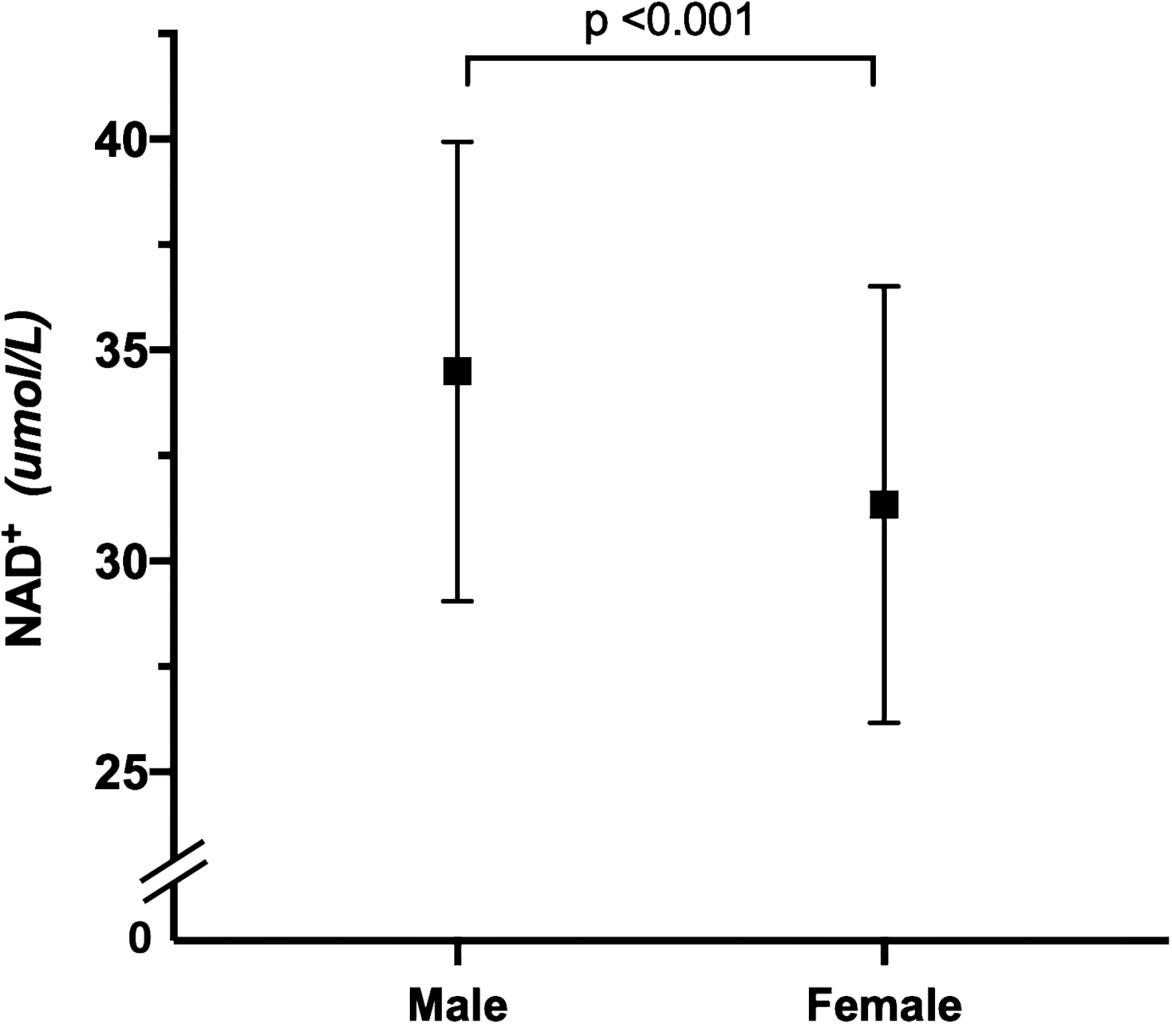
Gender difference in whole blood NAD^+^ contents. Comparison of whole blood NAD^+^ contents between men (N = 798) and women (N = 720). The dots and error bars represent mean and standard deviation of blood NAD^+^ contents in men and women, respectively.

**Figure 3:**
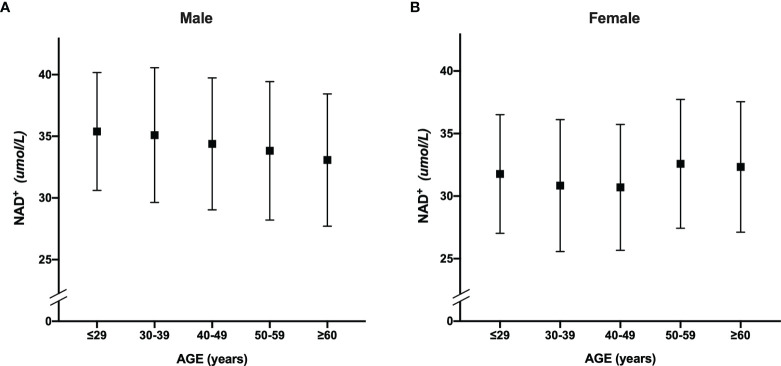
The trend of whole blood NAD^+^ contents with aging in men and women. **(A)** The trend of whole blood NAD^+^ contents with aging in males. **(B)** The trend of whole blood NAD^+^ contents with aging in females. The dots and error bars represent mean and standard deviation of blood NAD^+^ contents in five age groups, respectively.

**Figure 2** shows that the whole blood NAD^+^ contents of men (34.5 ± 5.4μmol/L) was significantly higher than women (31.3 ± 5.2 μmol/L) (p <0.001). The blood NAD^+^ contents show different trends with aging in men and women. The blood NAD^+^ decreased gradually with aging in men, while the female blood NAD^+^ showed a trend of fluctuations (**Figure 3**).

### Association of the Level of NAD^+^ and Aging in Total Participants

Crude and adjusted β coefficient with 95% CI of blood NAD^+^ contents with aging in total participants are presented in **Table 2**. Compared with ≤29 years group, adjusted β coefficient and 95% CI was −0.44 (−1.44, 0.55) for the 30–39 years group, −1.12 (−2.18, −0.06) for 40–49 years group, −0.67 (−1.78, 0.44) for 50–59 years group, and −1.02 (−2.34, 0.31) for ≥60 years group.

**Table 2:** Association of whole blood NAD^+^ contents with aging in total participants.

Age group (years)	N, %	β coefficient (95% CI)		
		Crude model	Adjust model	
**≤29**	132 (8.7)	Reference	Reference	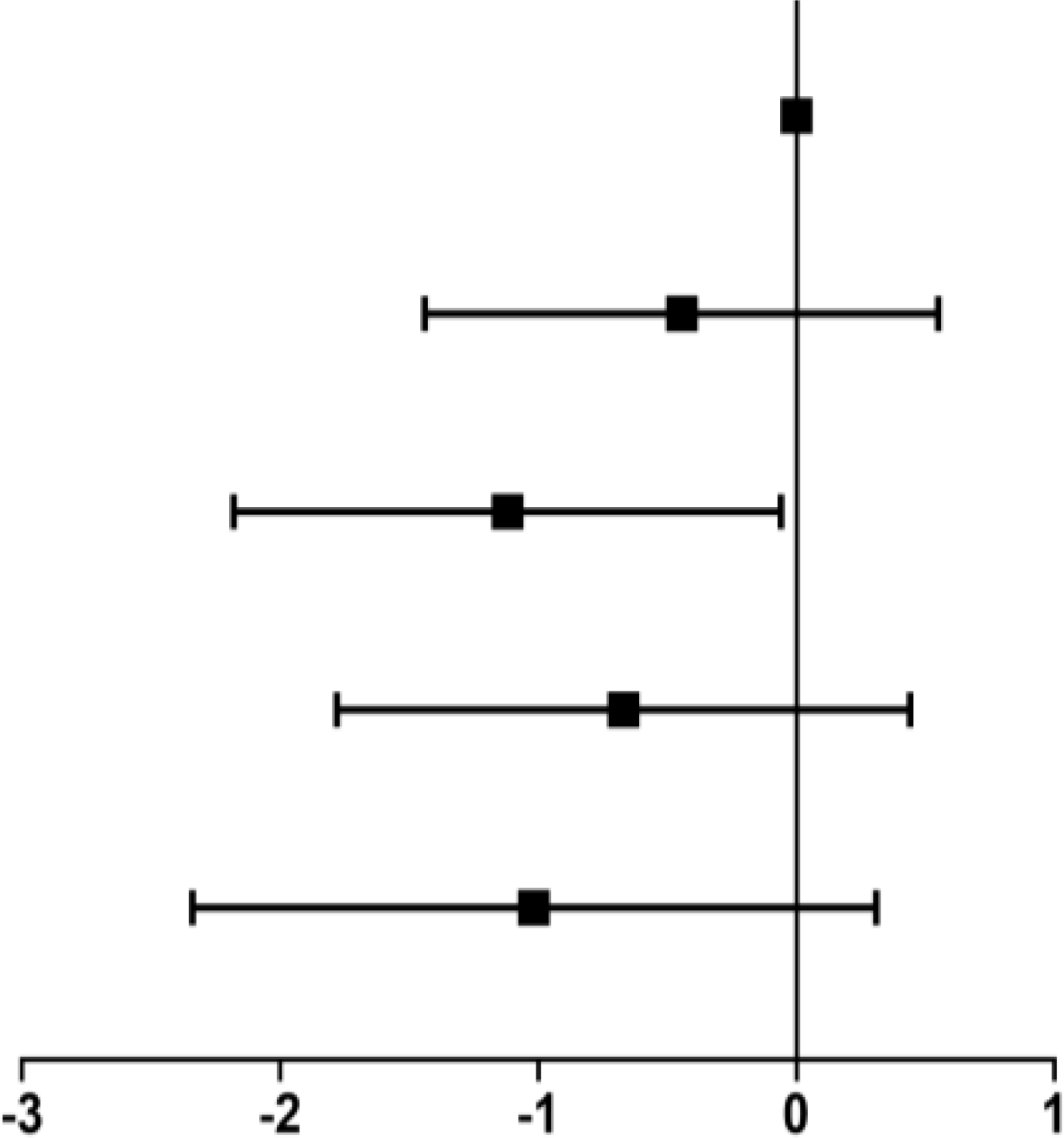
**30–39**	569 (37.5)	−0.19 (−1.25, 0.86)	−0.44 (−1.44, 0.55)	
**40–49**	377 (24.8)	−0.82 (−1.92, 0.28)	−1.12 (−2.18, −0.06)	
**50–59**	311 (20.5)	−0.02 (−1.15, 1.11)	−0.67 (−1.78, 0.44)	
**≥60**	129 (8.5)	−0.62 (−1.97, 0.72)	−1.02 (−2.34, 0.31)	

Adjustments made for sex, body mass index (BMI), smoking, drinking, history of hypertension, diabetes, hyperlipidemia, the number of RBC and the meat diet.

### Stratified Analysis by Gender

We performed gender-stratified analysis considering that the associations between blood NAD^+^ levels and aging were significantly altered by gender (P for interaction = 0.003). Crude and adjusted β coefficients with 95% CIs in the subgroups stratified by gender are reported in **Table 3**. In men, compared with ≤29 years group, the adjusted β coefficient and 95% CI was −0.27 (−1.80, 1.25) for 30–39 years group, −1.19 (−2.83, 0.46) for 40–49 years group, −1.47 (−3.16, 0.20) for 50–59 years group, and −2.16 (−4.16, −0.15) for ≥60 years group, which suggested an inverse association between aging and blood NAD^+^ contents in men with a significant level after 60 years. In women, the adjusted β coefficient and 95% CI was −1.15 (−0.22, 2.53) for ≤29 years group, 0.36 (−0.61, 1.32) for 30–39 years group, 1.57 (0.39, 2.75) for 50–59 years group, and 1.09 (−0.44, 2.63) for ≥60 years group with the 40–49 years as reference, which suggested there is no significant trend of NAD^+^ contents with aging in women, especially after 50 years.

**Table 3:** Stratified analysis by gender.

Age group (years)	N, %	β coefficient (95% CI)		
		Crude model	Adjust model	
**Men**				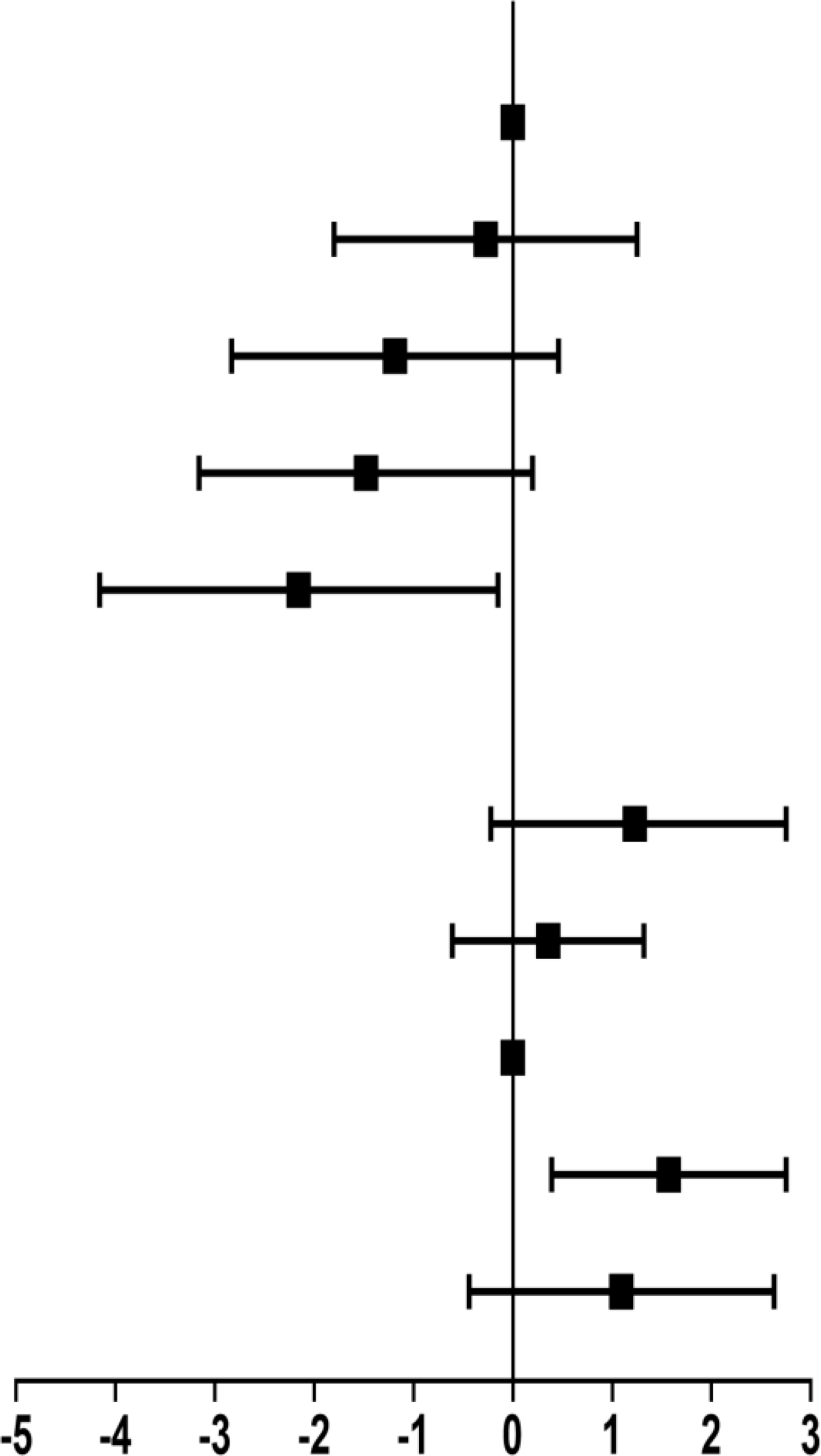
**≤29**	57 (7.1)	Reference	Reference	
**30–39**	307 (38.5)	−0.29 (−1.83, 1.24)	−0.27 (−1.80, 1.25)õ	
**40–49**	185 (23.2)	−1.00 (−2.61, 0.61)	−1.19 (−2.83, 0.46)	
**50–59**	183 (22.9)	−1.57 (−3.18, 0.04)	−1.47 (−3.16, 0.20)	
**≥60**	66 (8.3)	−2.31 (−4.24, -0.39)	−2.16 (−4.16, -0.15)	
**Women**				
**≤29**	75 (10.4)	1.07 (−0.30, 2.44)	1.15 (−0.22, 2.53)	
**30–39**	262 (26.4)	0.14 (−0.82, 1.09)	0.36 (−0.61, 1.32)	
**40–49**	192 (26.7)	Reference	Reference	
**50–59**	128 (17.8)	1.87 (0.73, 3.02)	1.57 (0.39, 2.75)	
**≥60**	63 (8.8)	1.63 (0.17, 3.09)	1.09 (−0.44, 2.63)	
**p for interaction**		<0.001	0.003	

Adjustments made for body mass index (BMI), smoking, drinking, history of hypertension, diabetes, hyperlipidemia, the number of RBC and the meat diet.

## Discussion

In our study, we observed a decline trend of NAD^+^ contents with aging before 50 years in total participants with significant level in the 40–49 years group. Nevertheless, this trend disappeared after the 50 years. In addition, we found the associations between blood NAD^+^ levels and aging were significantly altered by gender. The loss of blood NAD^+^ with aging was observed in men, especially in the middle-aged population. However, the whole blood NAD^+^ levels in women showed a trend of fluctuations after 50 years.

We did not observe a statistical difference of blood NAD^+^ level among five age groups in total population. There was not significant trend of NAD^+^ contents with aging in total participants. This result is comparable to other two studies (13, 22). However, we found that the blood NAD^+^ level in men is significantly higher than in women. In a previous study, Breton et al. had also reported that the gender difference of whole blood NAD^+^ contents, while there was no statistic difference due to the limitation of sample size (22). In addition, the plasma level of NAD^+^ in men had shown to be higher than women but without significant level (13). In our study, we expanded the sample size and this difference become statistically significant. In addition, the gender difference of NAD^+^ has been also observed in mice (19, 20).

We further addressed the levels of blood NAD^+^ among five age groups in both genders. We observed a significant interaction of gender and age for the changes of whole blood NAD^+^ levels. In men, we observed an inverse association of aging and whole blood NAD^+^ contents, especially in middle-aged population. The loss of NAD^+^ with aging might relate the imbalance of reduction-oxidation and DNA damage (23–25). In addition, a significant decrease in total NAD^+^ content was observed in tissues from adults with different age stage (30–50, 51–70, >71 years), as compared to newborns (0–1 years) (18). In women, the level of blood NAD^+^ firstly appeared to a minor decrease and then showed a trend of fluctuation after 50 years. This observation is consistent with the study of Breton that the significant difference between men and women in the correlation between blood NAD^+^ levels and age, and the decline trend only observed in men (22). We speculated this fluctuation trend in women after 50 years might influenced by sex hormones. The female menopause happened in 50 years around generally, and the sex hormones greatly altered in menopausal transition and postmenopausal period (26, 27). However, the relationship of NAD^+^ level and sex hormones are still sparse. Further investigation on the relationship of NAD^+^ contents and sex hormones need be conducted.

There were several limitations to this study. First, our present study analyzed the cross-sectional association between aging and blood NAD^+^ contents. Hence, no temporal relationship or causation between these two could be established from our study. Second, we only recruited the participants aged over 18 years so that we lack the direct comparisons with children, prepubescent teens and even newborns. This might partly explain the failure to observe a significant association between NAD^+^ contents and aging in other male age groups. In addition, the association of NAD^+^ with aging might varied across organs and tissues (28). We only tested the NAD^+^ level in whole blood samples so that it could reflect the association of aging and NAD^+^ contents in human whole blood. Moreover, the whole blood NAD^+^ is likely to reflect NAD^+^ levels in red blood cells. Further studies should be conducted to explore the association in other tissues or organs. Furthermore, data on the NAD^+^ precursors, such as free NA, NAM, NADH would give more information in the NAD^+^ related studies but were absent in the current study. In mammalian cells, there are two pyridine nucleotide pools: NADP(H) and NAD(H). Despite being structurally related, NAD(H) and NADP(H) are typically recognized by different enzymes and thus exhibit distinct functions (29, 30). NAD(H) primarily participates in catabolic reactions; however, NADP^+^ and its reduced counterpart, NADPH, are mainly involved in anabolic reactions and the anti-oxidative defense. Although the relatively independent, NADP(H) and NAD(H) pools are closely connected, with conversion of NAD^+^ into NADP^+^ by NAD kinases and the reversible reaction by NADP^+^ phosphatase such as by Nocturnin (NOCT) and MESH1 (31). In addition, when NAD is in transient shortage in cells, many NAD-dependent redox enzymes have an isoform that uses NADP instead of NAD such as IDH and ME (30). We will comprehensively and systematically study the relationship between coenzyme II-NADP metabolism and aging by measuring NADP, NADPH and their ratio in whole blood and intestinal microorganisms in the future study.

In summary, the association of whole blood NAD^+^ contents and aging differed in men and women in human to some extent. The loss of whole blood NAD^+^ with aging only observed in men, especially in male middle-aged population, while the NAD^+^ levels in women showed no significant difference among five age groups. Hence, it is crucial to consider the gender difference in NAD^+^ related studies in the future and further elucidate the mechanisms of NAD^+^ homeostasis between genders.

## Data Availability Statement

The original contributions presented in the study are included in the article/**Supplementary Material**, further inquiries can be directed to the corresponding authors.

## Ethics Statement

The studies involving human participants were reviewed and approved by the Ethical Committees of the Staff Hospital of Jidong Oilfield of China National Petroleum Corporation. The patients/participants provided their written informed consent to participate in this study.

## Author Contributions

YZ and ZYJ conceived and designed the study and analyses. FY, XD and YY analyzed data and drafted the paper. FFH collected and LL washed the data. XDC, XGZ, JPZ. and YGQ. performed the detection of blood NAD^+^. FX, YZZ, JG and XMX carried out the critical revision of the article. XGZ revised it critically for important intellectual content. FY, XD and YY contributed equally. All authors listed have made a substantial, direct, and intellectual contribution to the work and approved it for publication.

## Funding

This study was supported by the National Key R&D Program of China (2018YFC2000705 and 2021YFC2500500); the National Natural Science Foundation of China (Grant Nos. 81973112, 82001468, 92049304, and 92049302); the Nutritional Science Research Foundation of BYHEALTH Institute of Nutrition & Health; Grants of Postdoctoral Fund of the First Affiliated Hospital, Jinan University (809033); and the China Postdoctoral Science Foundation (2020M673064).

## Conflict of Interest

The authors declare that the research was conducted in the absence of any commercial or financial relationships that could be construed as a potential conflict of interest.

## Publisher’s Note

All claims expressed in this article are solely those of the authors and do not necessarily represent those of their affiliated organizations, or those of the publisher, the editors and the reviewers. Any product that may be evaluated in this article, or claim that may be made by its manufacturer, is not guaranteed or endorsed by the publisher.
