# Supplementary material for: Association of Human Whole Blood NAD+ Contents With Aging
Source: Front Endocrinol (Lausanne). 2022 Mar 21;13:829658. doi: 10.3389/fendo.2022.829658 (PMC8979162; doi:10.3389/fendo.2022.829658)
Supplement: Supplementary file 1 [file DataSheet_1.docx]

Supplementary Material

## Supplementary Figures

##
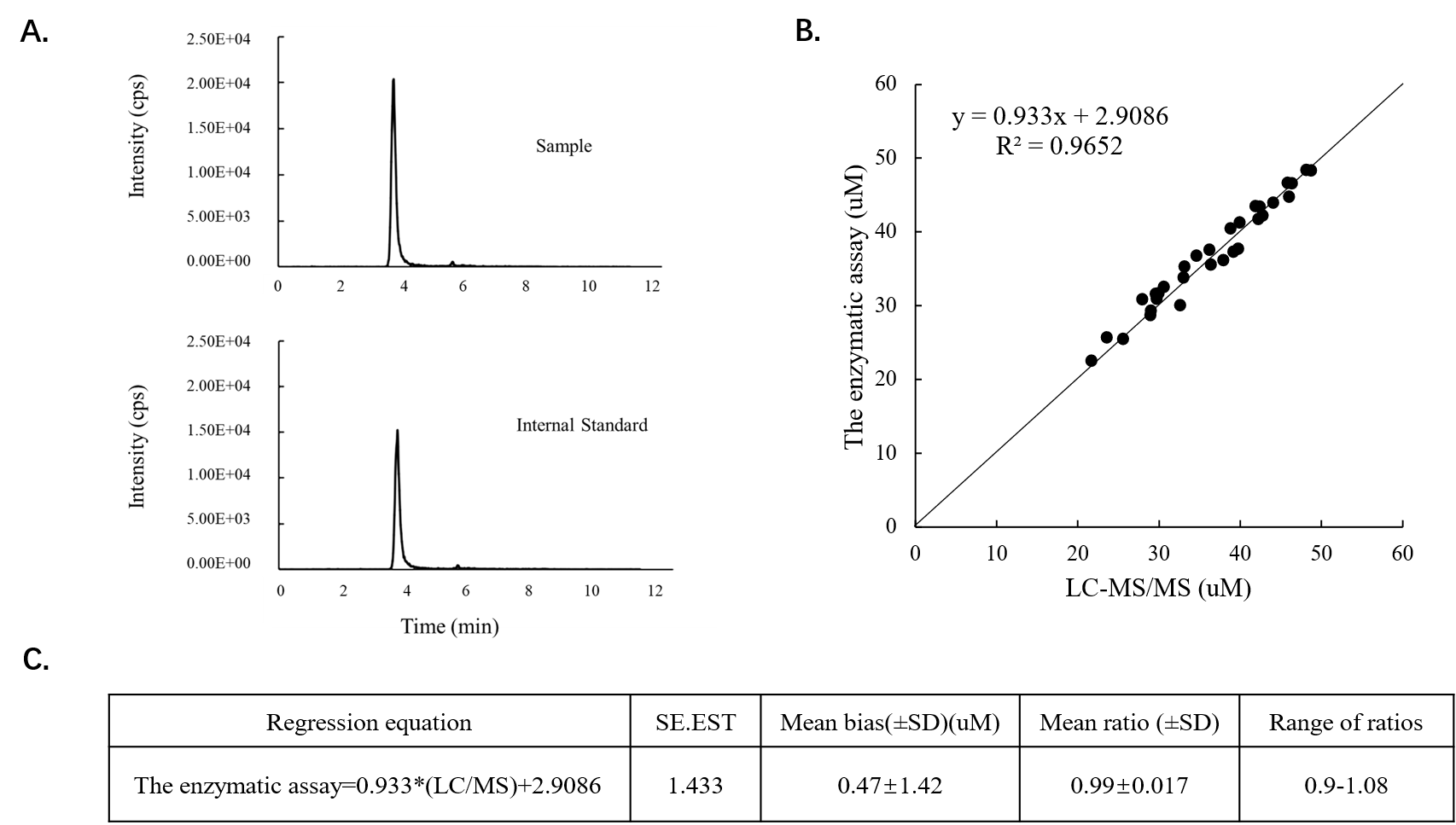


**Supplementary Figure 1.** Correlation between the enzymatic assay and LC-MS/MS (A.) There presentative chromatograms of NAD^+^ and 13C5-NAD^+^ in human blood. (B-C.) Blood concentrations measured by the enzymatic assay compared with the reference method (LC/MS/MS) in 31 adults.

**Supplementary Table 1. The characteristics influenced NAD^+^ contents.**

| Characteristics | Total | NAD^+^ (μmol/L) | | | | p value |
| --- | --- | --- | --- | --- | --- | --- |
|  |  | Quartile 1  (<29.4) | Quartile 2  (29.4~32.8) | Quartile 3  (32.8~36.4) | Quartile 4  (≥36.4) |  |
| N, % | 1518 | 375 (24.7) | 384 (25.3) | 380 (25.0) | 379 (25.0) |  |
| Age (years) | 43.0±11.3 | 43.4±10.8 | 43.8±12.0 | 42.4±11.2 | 42.4±11.0 | <0.001 |
| Age-Male | 43.5±11.3 | 46.1±11.7 | 45.7±11.6 | 42.3±11.0 | 41.8±10.6 | <0.001 |
| Age-Female | 42.5±11.2 | 42.2±10.1 | 42.2±12.1 | 42.6±11.5 | 43.9±11.9 | 0.57 |
| Males (N, %) | 798 (52.6) | 117 (31.2) | 180 (46.9) | 231 (60.8) | 270 (71.2) | <0.001 |
| RBC (10^12^/L) | 4.8±0.5 | 4.6±0.5 | 4.7±0.5 | 4.8±0.5 | 4.9±0.5 | <0.001 |
| Meat diet (N,%) |  |  |  |  |  | 0.01 |
| Never | 32 (2.11) | 17 (4.5) | 7 (1.8) | 5 (1.3) | 3 (0.8) |  |
| Occasionally | 1222 (80.5) | 296 (78.9) | 307 (80.0) | 301 (79.2) | 318 (83.9) |  |
| Very often | 264 (17.4) | 62 (16.5) | 70 (18.2) | 74 (19.5) | 58 (15.3) |  |
| Vegetable diet (N,%) |  |  |  |  |  | 0.81 |
| Never & Occasionally | 100 (6.6) | 28 (7.5) | 24 (6.3) | 22 (5.8) | 26 (6.9) |  |
| Very often | 1418 (93.4) | 347 (92.5) | 360 (93.8) | 358 (94.2) | 353 (93.1) |  |

Note: Values for categorical variables are given as number or number (percentage); Values for continuous variables are given as mean ± standard deviation.
